# The effects of a mitochondrial targeted peptide (elamipretide/SS31) on BAX recruitment and activation during apoptosis

**DOI:** 10.1186/s13104-021-05613-9

**Published:** 2021-05-22

**Authors:** Joshua A. Grosser, Rachel L. Fehrman, Dennis Keefe, Martin Redmon, Robert W. Nickells

**Affiliations:** 1 Department of Ophthalmology and Visual Sciences, University of Wisconsin-Madison, 571A Medical Sciences, 1300 University Avenue, Madison, WI 53706 USA; 2 Wake Forest University School of Medicine, Winston-Salem, NC USA; 3 Stealth BioTherapeutics Inc., Newton, MA USA; 4 McPherson Eye Research Institute, University of Wisconsin-Madison, Madison, WI USA

**Keywords:** BAX, Intrinsic apoptosis, Mitochondrial dysfunction, Mitochondrial outer membrane permeabilization, Mitochondrial fragmentation, Elamipretide (SS31)

## Abstract

**Objective:**

Elamipretide (SS31) is a mitochondria-targeted peptide that has reported functions of stabilizing mitochondrial cristae structure and improving mitochondrial bioenergetics. Several studies have documented cell protective features of this peptide, including impairment of intrinsic apoptosis by inhibiting the recruitment and activation of the pro-apoptotic BAX protein. We used live-cell imaging of ARPE-19 cells expressing fluorescently labeled BAX, cytochrome c, and a mitochondrial marker to investigate the effect of elamipretide on the kinetics of BAX recruitment, mitochondrial outer membrane permeabilization (as a function of cytochrome c release), and mitochondrial fragmentation, respectively.

**Result:**

In nucleofected and plated ARPE-19 cells, elamipretide accelerated the formation of larger mitochondria. In the presence of the apoptotic stimulator, staurosporine, cells treated with elamipretide exhibited moderately slower rates of BAX recruitment. Peptide treatment, however, did not significantly delay the onset of BAX recruitment or the final total amount of BAX that was recruited. Additionally, elamipretide showed no impairment or delay of cytochrome c release or mitochondrial fragmentation, two events associated with normal BAX activation during cell death. These results indicate that the protective effect of elamipretide is not at the level of BAX activity to induce pro-apoptotic mitochondrial dysfunction after the initiation of staurosporine-induced apoptosis.

**Supplementary Information:**

The online version contains supplementary material available at 10.1186/s13104-021-05613-9.

## Introduction

Intrinsic apoptosis is tightly regulated by mitochondrial dysfunction that is controlled by proteins of the BCL2 gene family [1], which are grouped on the basis of sharing BCL2 Homology (BH) domains. Pro-apoptotic BAX is a latent protein that predominantly resides in a globular conformation in the cytosol, but also in a state of equilibrium with the mitochondrial outer membrane (MOM). Anti-apoptotic BCL2 family proteins, such as BCL-X, function to shuttle BAX back into the cytosol under steady state conditions [2]. During apoptosis, BAX is recruited to the MOM, where it forms dimers and then large molecular weight oligomers. Kinetic studies show that the recruitment phase follows a sigmoidal function [3] and is completed in a period of approximately 20–30 min.

A principal function of BAX activation is the permeabilization of the MOM (MOMP) to allow the release of a variety of pro-apoptotic signaling molecules such as cytochrome c. Live cell imaging studies show that MOMP and the release of cytochrome c occurs nearly instantaneously with the initiation of BAX recruitment and before the formation of large oligomers [3, 4]. At the end of BAX recruitment mitochondria undergo dramatic fragmentation [5]. This process is significantly reduced in cells with impaired DRP1 function [6], but also in the presence of mutant BAX which has selectively impaired function during apoptosis [5]. The relationship between DRP1 and BAX centers around regulation of cardiolipin transport and cristae structure. In healthy cells, cardiolipins are predominantly localized to the mitochondrial inner membrane (MIM), where they help stabilize cristae. During apoptosis, cardiolipins are transferred to the MOM, destabilizing the cristae structure and promoting increased curvature of the MOM. Evidence suggests that DRP1 regulates the process of cristae destabilization [7]. Cristae destabilization also frees cytochrome c into the intermembrane space. BAX accumulation is favored at the sites of high cardiolipin density, prompting some interpretations that DRP1 is required to stimulate BAX recruitment to these regions [8]. Loss of DRP1, however, does not prevent the initiation and early recruitment of BAX [5], nor does it block MOMP and the release of SMAC/Diablo [9, 10], although cytochrome c release is impaired [7, 10]. In cells with impaired DRP1 function, BAX recruitment is limited to approximately 75% of the level found in normal cells [5].

The mitochondrial-targeted peptide elamipretide (SS31) has consistently been shown to ameliorate age-related conditions affecting muscle, cardiac, ocular, and nephrotic tissues [11–15] and has been shown to be protective in animal models of neurodegeneration [16–20]. A variety of factors could account for the beneficial effects of elamipretide including stabilization of mitochondrial cristae structure [15], modulation of lipid surface properties including charge [21], and improved mitochondrial function and transport [20]. Some reports suggest that elamipretide also impairs the recruitment of BAX to the mitochondria [17] and prevents mitochondrial fission [14] during apoptosis. Given the ability of elamipretide to associate with cardiolipins and to stabilize cristae structure [15], we hypothesized that elamipretide may mimic DRP-1 defects and alter BAX recruitment to the MOM and/or inhibit downstream events associated with BAX activation such as MOMP and mitochondrial fragmentation.

## Main text

### Methods

#### Cell culture and nucleofection

ARPE-19 cells (immortalized human adherent retinal pigmented epithelial cells) were a gift from Dr. Aparna Lakkaraju (University of California-San Francisco) and were used in all live-cell imaging experiments at passage 25. Cells were cultured in DMEM: F-12 (Hyclone, GE Healthcare Life Sciences, Marlborough, MA) supplemented with 10% FBS (Atlanta Biologicals, Norcross, GA) and 1% penicillin streptomycin (Cellgro Mediatech, Inc., Manassas, VA) at 37 °C and 5% CO_2_.

Plasmids used for these experiments were obtained as follows. MitoBFP and mCherry-BAX were cloned in the laboratory using vectors TagBFP-N (Evrogen, Moscow, Russia) and pmCherry-C1 (Clonetech, Mountainview, CA) as described previously [3]. Cytochrome c-GFP (Addgene #41182) and SMAC-GFP (Addgene #40881) were gifts from Douglas Green. All transgenes were run using the CMV immediate early promoter. For nucleofection, 10^6^ cells were combined with 3–5 µg of each plasmid and nucleofected using an Amaxa Nucleofector (Lonza, Basel, Switzerland). After nucleofection 3.5–5.0 × 10^5^ cells were plated on 35 mm glass bottomed (No. 1.5 optical grade) MatTek dishes (Ashland, MA) and allowed to recover for 16–24 h. Cells were then incubated with different concentrations of elamipretide (in ethanol, 1:2000 final dilution), and apoptosis was induced by the addition of staurosporine (STS) to a final concentration of 1 µM (DMSO 0.01% v/v). Vehicle treatment contained the same ethanol: DMSO levels. Cells were maintained in DMEM: F12 media (with serum) until ready for imaging. Imaging was conducted in imaging media without serum.

#### Live-cell imaging and analysis

Live cell imaging was performed on an Andor Revolution XD spinning disc confocal microscope (Andor, Belfast, Northern Ireland) as previously described [3, 5, 22]. Images (20–30 0.22 µm sections) of ARPE-19 undergoing apoptosis were taken every 2 min beginning 14–17 h post STS addition using a 100× oil emersion objective (numerical aperture = 1.49). All imaging was performed under the same laser intensity (20%), electron-multiplying gain, and exposure time (200 ms). Image analysis was conducted using IMARIS 9.2.1 software (Neuroscience package, Bitplane Inc., South Windsor, CT) as described previously [3, 5].

#### Statistical analysis

For comparison of two samples, we used an un-paired Student’s *t*-test assuming unequal variance. For comparison of multiple samples, we used a 1-way ANOVA. For comparison of data along two linear slopes, we used a paired Student’s *t*-test. For comparison of the frequency of imaged cells that exhibited BAX recruitment as a function of time we used a Chi^2^ test. Statistical significance was set to alpha = 0.05.

### Results

#### Elamipretide accelerates an increase in mitochondrial volume in cultured ARPE-19 cells

To validate that elamipretide was influencing mitochondrial dynamics in ARPE-19 cells, they were first nucleofected with a plasmid expressing mitoBFP and cultured for 24 h. Cells were then incubated with 1 µM elamipretide or vehicle for 20 h (Additional file 1: Figure S1). At time points during the experiment, cells were imaged by confocal microscopy. Z-stack files were then analyzed with Imaris and the average volume of individual mitochondria was measured. Within 1 h, cells in both groups exhibited similarly low mitochondrial volumes. By 4 and 7 h, however, cells exposed to elamipretide exhibited a significant increase in mitochondrial volume (P = 0.005 and P = 0.002, respectively) relative to untreated cells. By 20 h, both groups of cells exhibited statistically similar volume increases (P = 0.207) that were substantially greater than those measured 1 h after plating.

#### Elamipretide does not delay the onset of BAX recruitment or MOMP

**Fig. 1:**
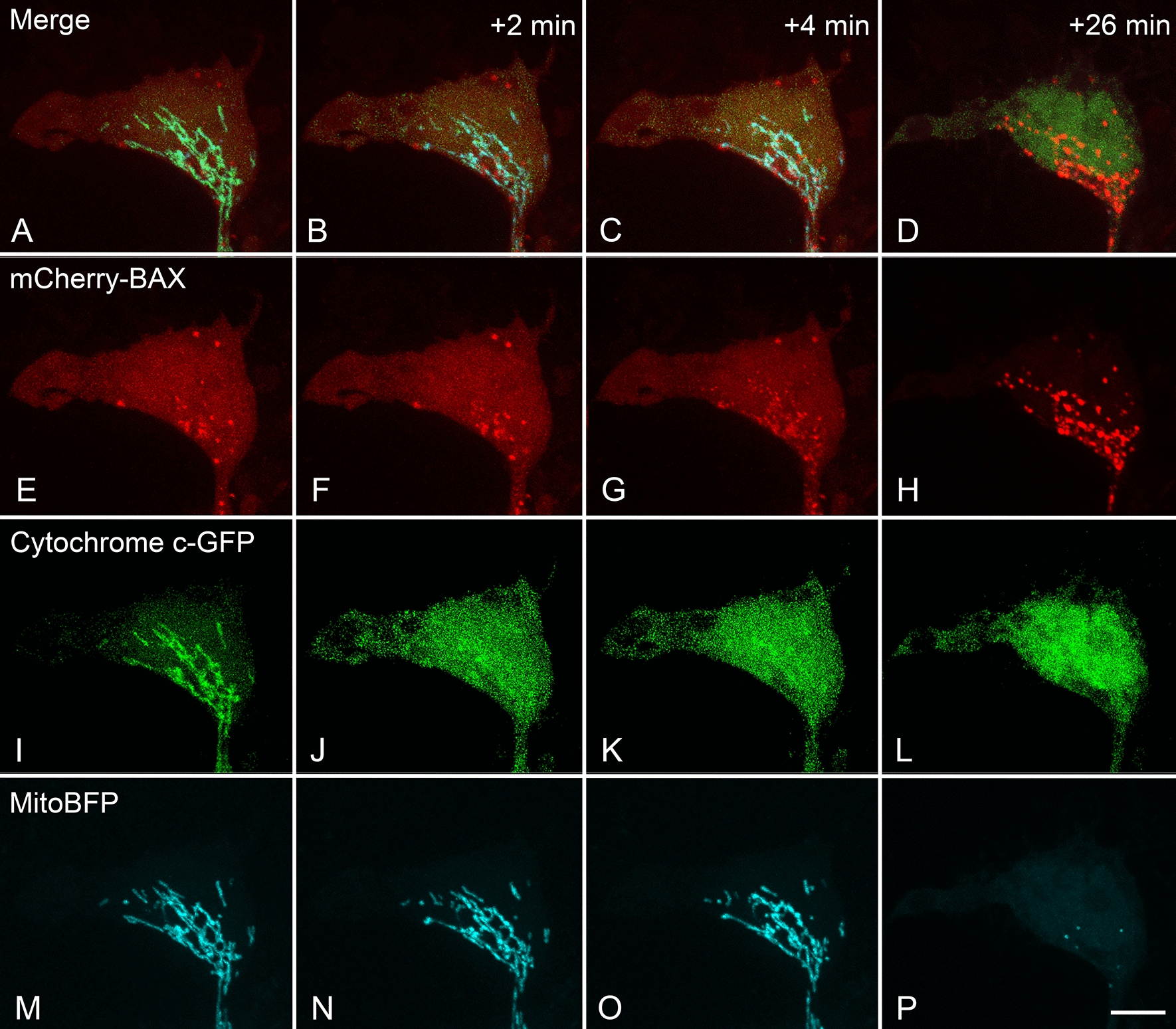
Time lapse imaging stills of an ARPE-19 cell undergoing apoptosis. The stills shown are of a cell treated with 0.01 µM elamipretide and 1 µM staurosporine. This cell was nucleofected with plasmids expressing mCherry-BAX, cytochrome c-GFP, and mitoBFP fusion proteins. The images just prior to the initiation of BAX recruitment are shown (**A**, **E**, **I**, **M**), along with +2 min, + 4 min, and + 26 min after this time stamp. Some BAX puncta are evident just prior to the declaration of BAX recruitment (**E**) and it is not clear if these are activated BAX aggregates, or excess fusion protein that is sequestered in lysosomes [3]. Once BAX recruitment is initiated, cytochrome c moves from a mitochondrial localization (**I**) to a diffuse cytosolic localization (**J**–**L**). Mitochondria remain intact through the initial stages of BAX recruitment (**M**–**O**) but become rapidly fragmented and difficult to detect shortly after BAX becomes fully recruited (**D**, **H**, **L**, P). These results are typical for both vehicle and elamipretide treated cells. Images for cytochrome c-GFP have been modified to enhance exposure levels. Size bar = 7 µm

ARPE-19 cells were triple nucleofected with plasmids for CMV-mCherry-BAX, CMV-cytochrome c-GFP, and CMV-mitoBFP. After 24 h, fresh media was added containing vehicle or different concentrations of elamipretide ranging from 0.01 to 10 µM. Apoptosis was induced by the addition of STS to a final concentration of 1 µM (considered time 0 min). All of the cells treated with elamipretide and STS showed the capacity to recruit and activate BAX leading to MOMP (Fig. 1). BAX recruitment was evident by the organization of labeled BAX protein moving from a diffuse cytosolic localization to form bright puncta that co-localized with mitoBFP labeled mitochondria. Additionally, 100% of the cells showing BAX translocation exhibited the release of cytochrome c-GFP (Fig. 1) that occurred rapidly at the time of initial BAX recruitment. The rapid release of these intramitochondrial proteins was also observed in vehicle treated ARPE-19 cells (Additional file 2: Figure S2) and is a typical feature of other cell types treated with STS [3].

These data showing the uninhibited capacity for BAX to be recruited to the MOM are inconsistent with a previous report showing reduced BAX accumulation to mitochondria [17]. It is possible that these conflicting observations are a result of a delay in the onset of BAX recruitment induced by the drug. To assess this, we mapped the timing of when BAX recruitment was initiated in all the cells that were imaged (Additional file 3: Figure S3). Imaging experiments were biased to an interval of 14–21 h when a majority of ARPE-19 exhibit apoptosis after STS addition. To evaluate if elamipretide treated cells were more or less likely to recruit BAX during this period, we used a Chi^2^ statistic to test if there was a difference in the proportion of cells that were imaged that converted, relative to imaged cells that failed to convert, during this window. There was no significant difference between any of the treatment groups compared to vehicle treated cells, or between the combined data of the treatment groups compared to vehicle (Additional file 4: Table S1).

#### Elamipretide retards the rate of BAX recruitment during apoptosis

**Fig. 2:**
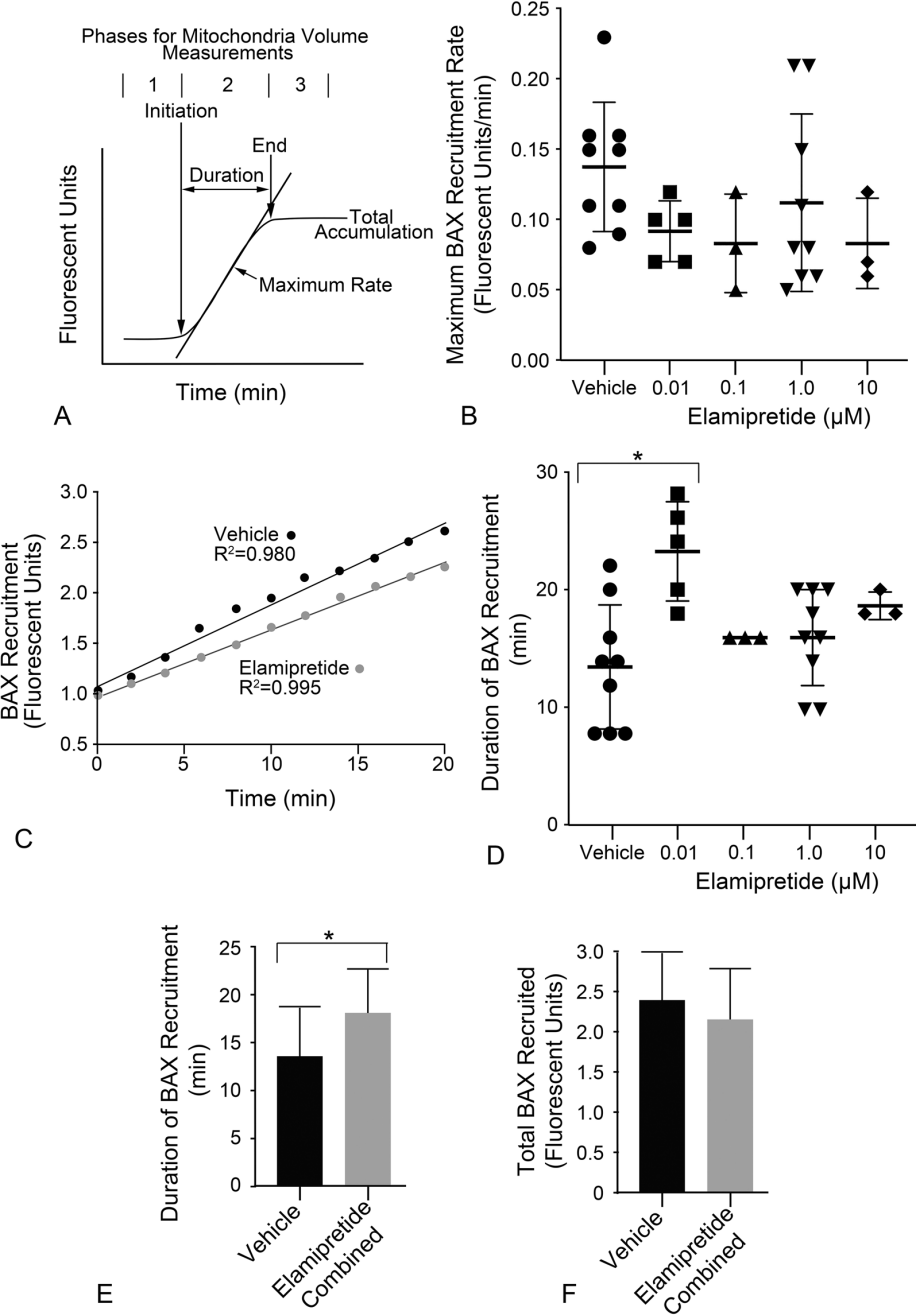
Kinetic analysis of mCherry-BAX recruitment in the presence of elamipretide. **A** A typical sigmoid BAX recruitment curve showing various metrics that were obtained, including the maximum rate and the times for initiation and end of BAX accumulation, which are used to determine the duration of the recruitment period. The plateau of the recruitment curve is used to determine the total accumulation of BAX being recruited. This curve represents individual BAX puntae, which are then averaged for the entire cell being examined. **B** Scatter plot of calculated maximal recruitment rates for cells analyzed in each treatment group. **C** Combined BAX recruitment data for vehicle (black circles) plotted against all elamipretide groups combined (grey circles). The data used in this analysis represents the first 20 min of recruitment for all cells that were successfully imaged and analyzed. The combined data for elamipretide shows a significantly slower rate of recruitment (P = 6.76e−5, paired *t*-test). **D** Duration periods for all treatment groups. Treatment with 0.01 µM elamipretide yielded significantly longer duration periods compared to vehicle treated cells (*P = 0.004). **E** Bar graph showing duration times of vehicle treated cells against the combined elamipretide treated groups. Durations are significantly longer in the presence of elamipretide (*P = 0.036). **F** Bar graph showing the total amount of BAX recruited in vehicle treated cells relative to the combined elamipretide treated groups. No significant difference was detected

Live cell imaging data was also analyzed to determine the maximum rate of BAX recruitment, the duration time for recruitment, and the total amount of BAX finally recruited (Fig. 2A). Scatter plots of the mean BAX recruitment rates for all cells are shown in Fig. 2B. No elamipretide group exhibited a significant reduction in rate compared to vehicle treated cells, although there was a trend to lower maximal rates in the presence of elamipretide. The data was reanalyzed to combine all treatment groups during a normalized 20 min period of recruitment and compared to the same period for vehicle treated cells. In a paired analysis elamipretide was shown to yield a significantly slower rate of BAX recruitment (Fig. 2C; P = 6.76e−5). Assessment of the duration of the recruitment period showed a trend for longer durations in elamipretide treated cells, with 0.01 µM elamipretide yielding significantly longer times relative to vehicle treated cells (Fig. 2D; P = 0.004) by an average of 9.6 min. When the elamipretide data was combined, the overall average duration time was 4 min longer for elamipretide cells compared to vehicle (Fig. 2E; P = 0.036). The moderate decrease in the rate of BAX recruitment, combined with the moderate increase in the duration time of recruitment resulted in no significant difference in the final amount of BAX that was recruited to cells under these conditions (Fig. 2F).

#### Elamipretide does not impact mitochondrial fragmentation after completion of BAX recruitment

**Fig. 3:**
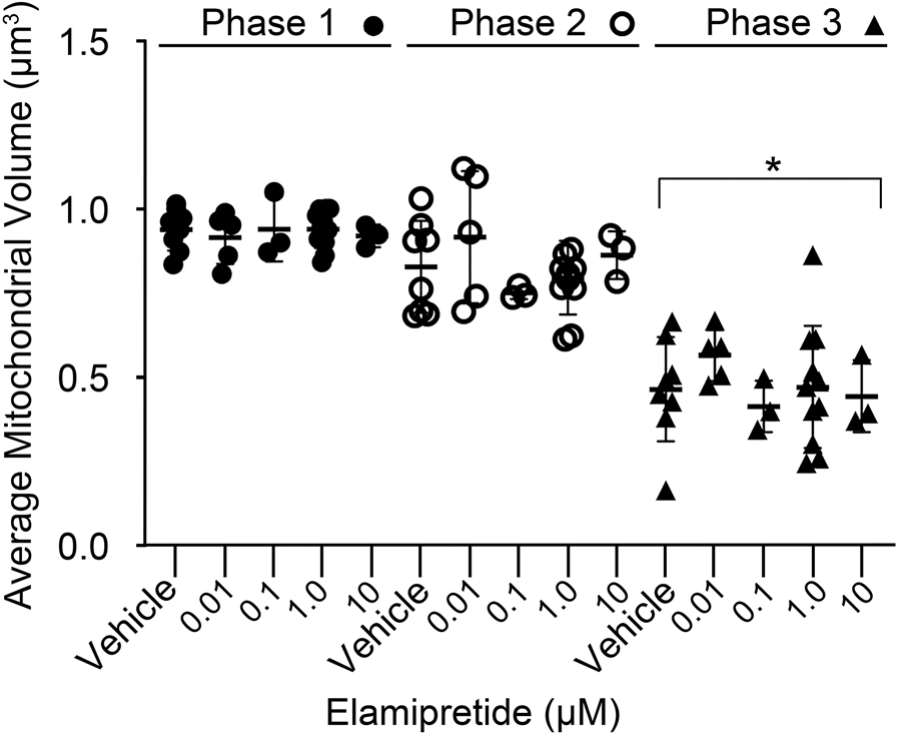
Analysis of apoptotic mitochondrial fragmentation in the presence of elamipretide. Scatter plot of average mitochondrial volume of ARPE-19 cells treated with vehicle or different concentrations of elamipretide. Mitochondrial volumes were calculated from time lapse confocal images of cells expressing mitoBFP to label mitochondria. Average volumes were calculated at least 20 min prior to the onset of mCherry-BAX recruitment (Phase 1), during the period of mCherry-BAX recruitment (Phase 2), and during a 20 min interval after mCherry-BAX recruitment (Phase 3) (see Fig. 2A). ANOVA analysis showed no difference in volumes among the treatment groups in each phase (P ≥ 0.38). All treatment groups showed a significant decrease in mitochondrial volume in Phase 3 relative to the other Phases (1-way ANOVA *P < 0.006)

To assess if elamipretide also interfered with mitochondrial fragmentation, we measured the average volume of mitochondria in cells in the periods before, during, and after the recruitment of BAX in ARPE-19 cells (Fig. 3, refer to Fig. 2A). There was no difference between treatment groups and vehicle treated cells in mitochondrial volume either before or during the period of BAX recruitment (ANOVA, P = 0.942 and P = 0.384, respectively). All groups showed a similar and significant decrease in mitochondrial volume following BAX recruitment (P < 0.006).

### Discussion

ARPE-19 cells exhibited small mitochondria 24 h after nucleofection and plating which increased in size over the course of then next 20 h regardless of treatment. This change was accelerated by elamipretide consistent with reports that it enhances mitochondrial dynamics [23], stabilizes cristae structure, and improves respiratory capacity [15]. Once at equilibrium, elamipretide is reported to not affect mitochondria in normal healthy cells [15].

Elamipretide is known to stabilize cardiolipin content and peroxidation levels in the MIM [15]. Additionally, DRP1 functions to regulate the disorganization of cristae and the transfer of cardiolipins to the MOM during apoptosis to create a lipid signaling platform that is conducive to the recruitment and organization of BAX [7]. We hypothesized that elamipretide would phenocopy the effects of DRP1 loss of function, which results in reduced BAX recruitment, suppression of cytochrome c release and mitochondrial fragmentation [5]. Elamipretide failed to induce any of these effects in our experiments. These results imply that elamipretide works by a mechanism independent from DRP1, which is consistent with studies showing that elamipretide and the DRP1 inhibitor Mdivi1 work synergistically to reduce mitochondrial dysfunction [18].

The mechanism of elamipretide on slowing the rate of BAX recruitment is not known. One possibility is that elamipretide moderately reduces cardiolipin transfer from the MIM to the MOM, thereby making the lipid signaling platform in the MOM less conducive to BAX integration. This hypothesis remains to be tested.

## Limitations

These studies were done on tissue culture cells treated with staurosporine, which may not reflect the apoptotic signaling of cells in a complex tissue. Additionally, cells were not synchronized prior to treatment and it is not clear how phases of the cell cycle might influence the activation of apoptosis.

## Supplementary Information

**Additional file 1: Figure S1.** Elamipretide stimulates a more rapid increase in mitochondrial volume. Mitochondria were identified in ARPE-19 cells nucleofected with a plasmid carrying a mitoBFP fusion protein. Nucelofected cells were plated in chamber slides, allowed to incubate for 24 h and then imaged for another 20 h. (A) Confocal image of a cell imaged at 1 h and then again at 7 h (B) after exposure to 1 µM elamipretide. Only the BFP channel is shown. Over time, mitochondria appear more filamentous and elongated. Size bar = 10 µm. (C) Quantification of average mitochondrial volumes of cells taken from both time-lapse and static images. The scatterplot shows data collected from individual cells at each time point (mean ± SD also indicated). Mitochondrial volume was measured in 3D reconstructions of confocal images using Imaris 9.2 imaging software. Elamipretide treated cells exhibit significantly greater mitochondrial volumes at 4 and 7 h after exposure to the peptide (*P = 0.005 and **P = 0.002, respectively). By 20 h, both treatment groups exhibit similarly larger mitochondria (P = 0.207)

**Additional file 2: Figure S2. ** Time lapse images of a vehicle-treated ARPE-19 cell undergoing apoptosis. In this example, SMAC-GFP was used to show mitochondrial outer membrane permeabilization. Size bar = 7 µm.

**Additional file 3: Figure S3.** Temporal assessment of cells undergoing mCherry-BAX recruitment during live-cell imaging experiments. Data collected from 13 imaging experiments are graphed. Each point represents a single cell. The horizontal lines indicate the duration of the imaging session and the points on each line indicate cells that underwent BAX recruitment during that session. Points represented in the column labeled “Other” represent cells that were set up for imaging but did not convert cytosolic BAX to punctate BAX.

**Additional file 4: Table S1.** Elamipretide does not delay the onset of BAX recruitment.

## Data Availability

Raw data, including imaging files, and reagents described in this study will be made available upon request to the corresponding author. Elamipretide was provided by Stealth BioTherapeutics, Inc., and requests for that reagent should be made directly to the company.

## Abbreviations

BH and BH3: BCL2 homology domain, and BCL2 homology domain 3
MIM: Mitochondrial inner membrane
MOM: Mitochondrial outer membrane
MOMP: Mitochondrial outer membrane permeabilization
STS: Staurosporine
