# Supplementary figures and images for: The effects of a mitochondrial targeted peptide (elamipretide/SS31) on BAX recruitment and activation during apoptosis
Source: BMC Res Notes. 2021 May 22;14:198. doi: 10.1186/s13104-021-05613-9 (PMC8141144; doi:10.1186/s13104-021-05613-9)

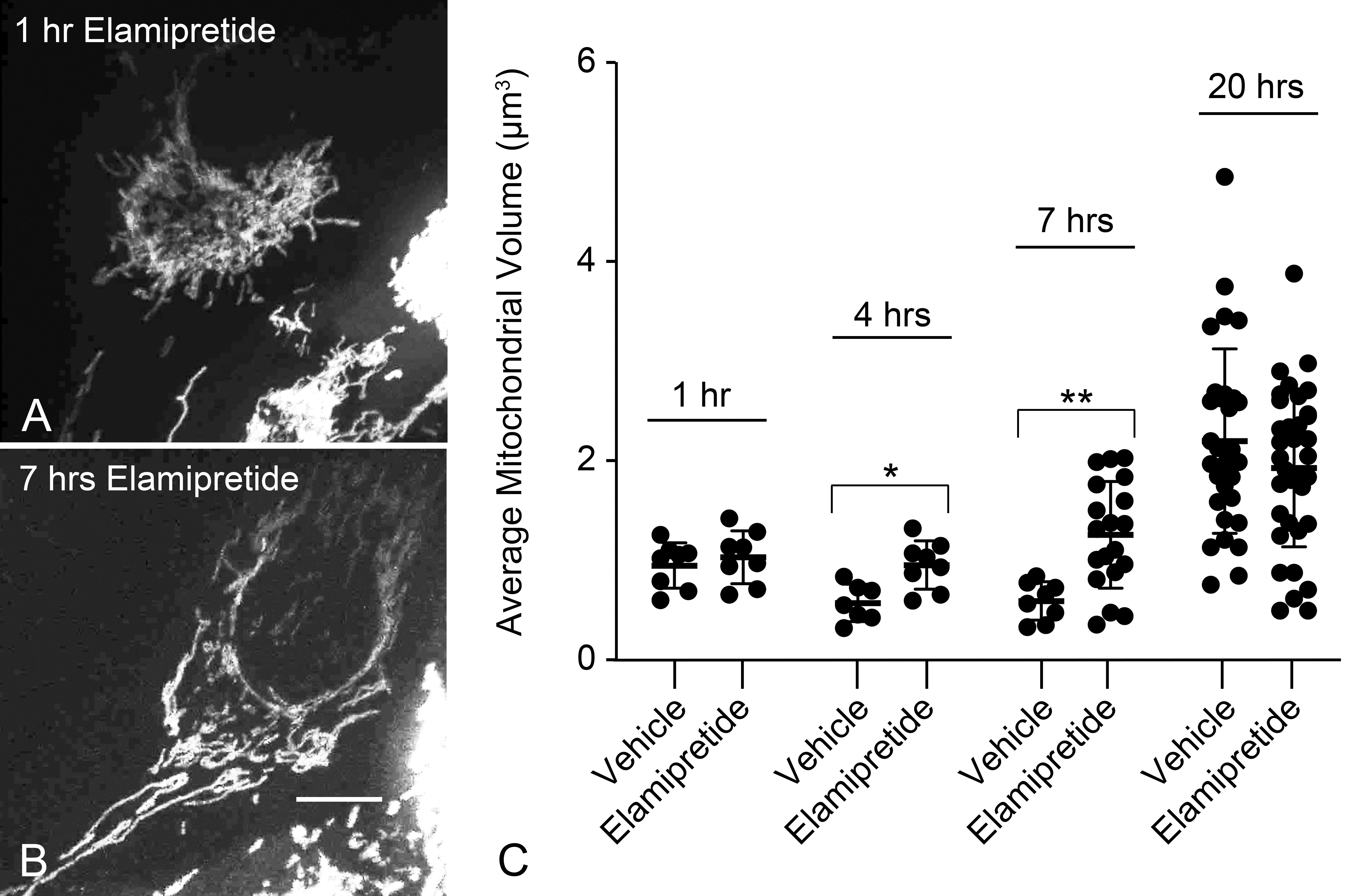

Supplement: Supplementary file 1 — Additional file 1: Figure S1. Elamipretide stimulates a more rapid increase in mitochondrial volume. Mitochondria were identified in ARPE-19 cells nucleofected with a plasmid carrying a mitoBFP fusion protein. Nucelofected cells were plated in chamber slides, allowed to incubate for 24 h and then imaged for another 20 h. (A) Confocal image of a cell imaged at 1 h and then again at 7 h (B) after exposure to 1 µM elamipretide. Only the BFP channel is shown. Over time, mitochondria appear more filamentous and elongated. Size bar = 10 µm. (C) Quantification of average mitochondrial volumes of cells taken from both time-lapse and static images. The scatterplot shows data collected from individual cells at each time point (mean ± SD also indicated). Mitochondrial volume was measured in 3D reconstructions of confocal images using Imaris 9.2 imaging software. Elamipretide treated cells exhibit significantly greater mitochondrial volumes at 4 and 7 h after exposure to the peptide (*P = 0.005 and **P = 0.002, respectively). By 20 h, both treatment groups exhibit similarly larger mitochondria (P = 0.207) [file 13104_2021_5613_MOESM1_ESM.jpg]

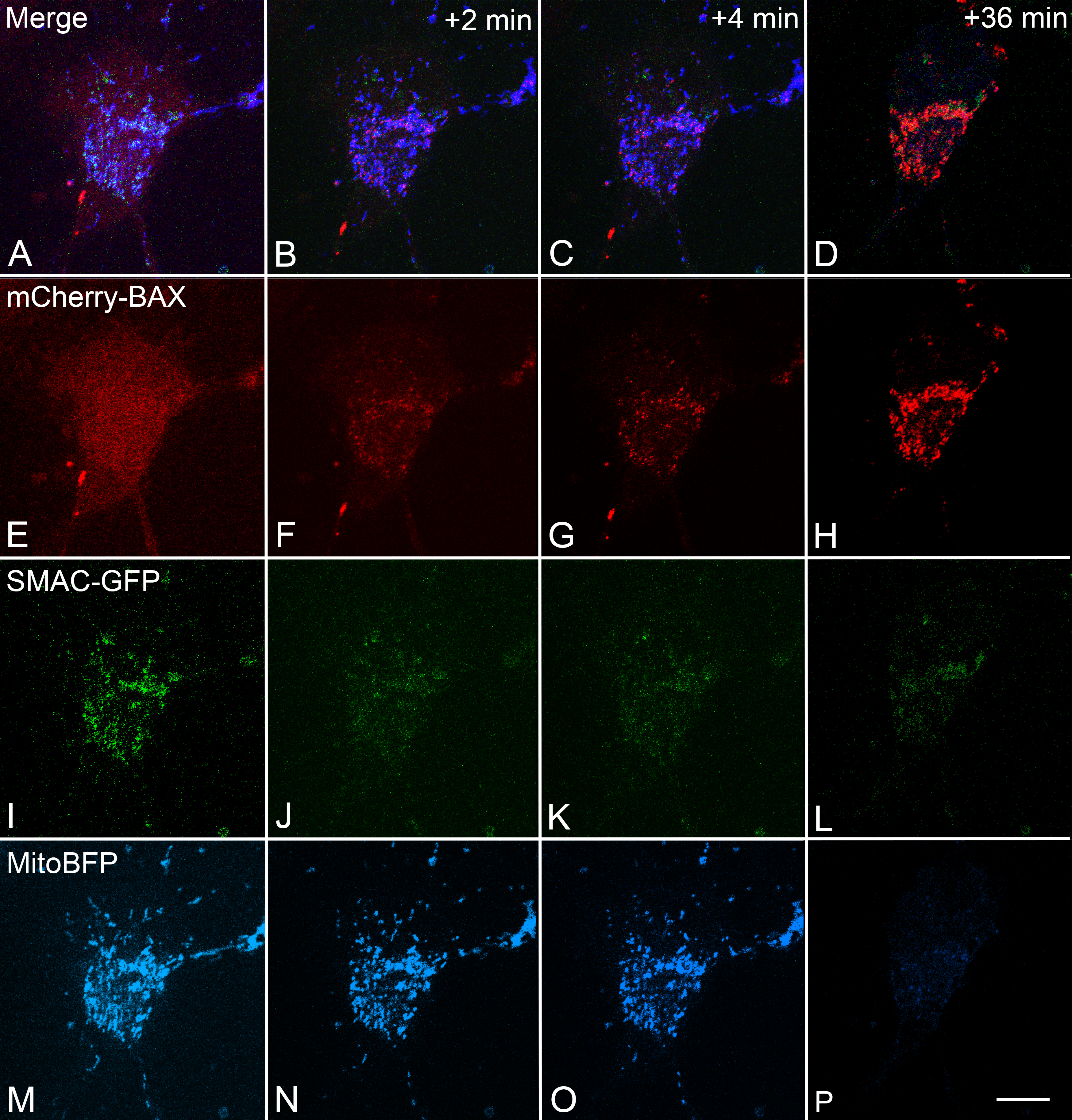

Supplement: Supplementary file 2 — Additional file 2: Figure S2. Time lapse images of a vehicle-treated ARPE-19 cell undergoing apoptosis. In this example, SMAC-GFP was used to show mitochondrial outer membrane permeabilization. Size bar = 7 µm. [file 13104_2021_5613_MOESM2_ESM.jpg]

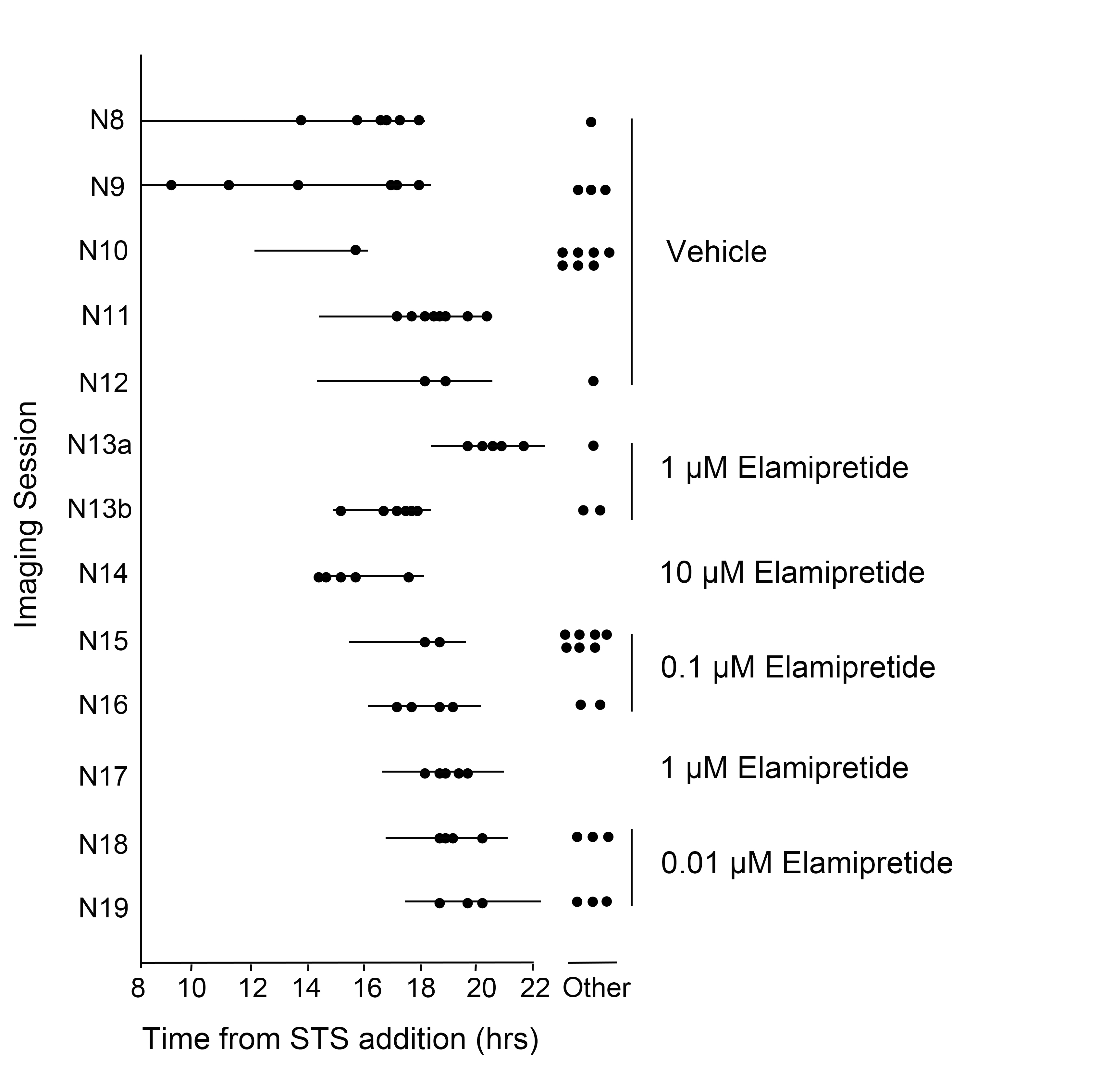

Supplement: Supplementary file 3 — Additional file 3: Figure S3. Temporal assessment of cells undergoing mCherry-BAX recruitment during live-cell imaging experiments. Data collected from 13 imaging experiments are graphed. Each point represents a single cell. The horizontal lines indicate the duration of the imaging session and the points on each line indicate cells that underwent BAX recruitment during that session. Points represented in the column labeled “Other” represent cells that were set up for imaging but did not convert cytosolic BAX to punctate BAX. [file 13104_2021_5613_MOESM3_ESM.jpg]
