# Supplementary material for: The effects of a mitochondrial targeted peptide (elamipretide/SS31) on BAX recruitment and activation during apoptosis
Source: BMC Res Notes. 2021 May 22;14:198. doi: 10.1186/s13104-021-05613-9 (PMC8141144; doi:10.1186/s13104-021-05613-9)
Supplement: Supplementary file 4 — Additional file 4: Table S1. Elamipretide does not delay the onset of BAX recruitment. [file 13104_2021_5613_MOESM4_ESM.docx]

| Treatment | Converting Cells in Imaging Window* | Non-converting Cells in Imaging Window** | Chi^2^ Statistic | P value |
| --- | --- | --- | --- | --- |
| Vehicle | 21 | 11 |  |  |
| 0.01 µM Elamipretide | 7 | 6 | 0.546 | 0.460 |
| 0.1 µM Elamipretide | 6 | 9 | 2.743 | 0.098 |
| 1.0 µM Elamipretide | 15 | 4 | 1.019 | 0.313 |
| 10 µM Elamipretide | 5 | 0 | 0.733† | 0.392 |
| Elamipretide Combined | 33 | 19 | 0.040 | 0.841 |
